# Risk factors and incidence of mediastinitis in patients with lung abscess and sepsis

**DOI:** 10.1186/cc14011

**Published:** 2014-12-03

**Authors:** E Shalaeva, B Babadjanov, B Janabaev, A Shalaeva

**Affiliations:** 1 Republican Center of Purulent Surgery and Complications of Diabetes, Tashkent Medical Academy, Tashkent, Uzbekistan

## Introduction

Mediastinitis is a life-threatening condition, which is accompanied by high rates of mortality in cases of delayed diagnosis and inadequate treatment. The aim of the study was to identify the risk factors and incidence of mediastinitis in patients with lung abscess and sepsis.

## Methods

In 2013, 218 consecutive patients (83 women and 135 men) with lung abscess and sepsis aged 45.8 ± 13.2 years were operated. They had a full range of laboratory and instrumental examinations, including echocardiography and computed tomography.

## Results

Aerobic-anaerobic association in sputum was revealed in all patients with lung abscess and sepsis, *Candida *spp. in 34 (15.6%). Blood culture was positive only in 59 (27%) patients, which had not previously received antibacterial therapy (polymicrobial flora including *Staphylococcus *and *Streptococcus *specimen). Empyema was diagnosed in 123 patients (56.4%), 31 (14.2%) of them were complicated by mediastinitis. The main clinical symptoms of mediastinitis were hyperthermia (100%), dysphagia (83.8%), dyspnea (80.6%), chest pain (61.3%), orthopnea (61.3%), and tachycardia (87.1%). The computer tomography revealed an increase in mediastinum size with accumulation of fluids and fluid in the pleural cavities (100%), free gas in the mediastinum (45.1%), enlarged mediastinal lymph nodes (45.1%), and fluid in the pericardium cavity (48.4%). To analyze the risk factors, we include 31 patients with lung abscess and sepsis complicated by mediastinitis in the first group, and 187 patients without mediastenitis in the second group. Groups were similar in age (46.1 ± 8.2 years vs. 45.8 ± 13.2 years). A total 77.4% of patients with mediastinitis were women who suffered from type 2 diabetes (HbA1c = 9.7 ± 1.4%), congestive heart failure and anemia. Significant differences in the groups according to the data of laboratory and instrumental studies are presented in Table 1.

**Table 1:** Risk factors for mediastinitis in patients with lung abscess and sepsis

	With mediastinitis (*n *= 31)	Without mediastinitis (*n *= 187)	*P *value
Gender, male/female, *n *(%)	7 (22.6)/24 (77.4)	128 (68.5)/59 (31.5)	0.001
Type 2 diabetes mellitus, *n *(%)	26 (83.9)	42 (22.5)	0.001
Body mass index	32.3 ± 5.3	27.8 ± 6.1	0.031
Hemoglobin (g/l)	87.9 ± 9.2	128.4 ± 18.4	< 0.001
Fibrinogen (mg%)	800 ± 200	533 ± 166	< 0.001
End-diastolic volume of the left ventricle (ml)	139 ± 27	108 ± 28	0.024
End-systolic volume of the left ventricle (ml)	66.1 ± 8.2	46.8 ± 10.4	< 0.001
Left ventricular ejection fraction (%)	52.4 ± 2.2	57.2 ± 3.4	0.002

## Conclusion

In total, 14.2% of patients presented with lung abscess and sepsis complicated by mediastinitis, more commonly in women with diabetes mellitus, obesity, anemia and reduced ejection fraction of the left ventricle.

